# Therapeutics Targeting Skeletal Muscle in Amyotrophic Lateral Sclerosis

**DOI:** 10.3390/biom14070878

**Published:** 2024-07-22

**Authors:** Jinghui Gao, Elijah Sterling, Rachel Hankin, Aria Sikal, Yao Yao

**Affiliations:** Regenerative Bioscience Center, Department of Animal and Dairy Science, College of Agricultural and Environmental Science, University of Georgia, Athens, GA 30602, USA; jinghui.gao@uga.edu (J.G.); elijah.sterling@uga.edu (E.S.); rachel.hankin@uga.edu (R.H.); aria.sikal@uga.edu (A.S.)

**Keywords:** amyotrophic lateral sclerosis (ALS), skeletal muscle, muscle satellite cells (MuSCs), inflammation, neuromuscular junction (NMJ)

## Abstract

Amyotrophic lateral sclerosis (ALS) is a complex neuromuscular disease characterized by progressive motor neuron degeneration, neuromuscular junction dismantling, and muscle wasting. The pathological and therapeutic studies of ALS have long been neurocentric. However, recent insights have highlighted the significance of peripheral tissue, particularly skeletal muscle, in disease pathology and treatment. This is evidenced by restricted ALS-like muscle atrophy, which can retrogradely induce neuromuscular junction and motor neuron degeneration. Moreover, therapeutics targeting skeletal muscles can effectively decelerate disease progression by modulating muscle satellite cells for muscle repair, suppressing inflammation, and promoting the recovery or regeneration of the neuromuscular junction. This review summarizes and discusses therapeutic strategies targeting skeletal muscles for ALS treatment. It aims to provide a comprehensive reference for the development of novel therapeutics targeting skeletal muscles, potentially ameliorating the progression of ALS.

## 1. Introduction

Amyotrophic lateral sclerosis (ALS) is a devastating neuromuscular disorder characterized by progressive degeneration of motor neurons (MNs) and severe muscle atrophy. Most ALS cases are sporadic, and a minority of cases (5–10%) are familial [1]. Over 30 genes have been identified as being linked to ALS [2], with the most prevalent mutations found in *SOD1* (Cu/Zn superoxide dismutase 1), *C9ORF72* (chromosome 9 open reading frame 72), *TARDBP* (TAR DNA-binding protein 43; *TDP-43*), and *FUS* (fused in sarcoma) [3]. ALS patients endure progressive muscle wasting, which gradually impairs their ability to move, speak, eat, and breathe. Typically, patients succumb to respiratory failure within 2 to 5 years after diagnosis [4,5]. No efficacious therapies have been developed, mainly due to an elusive pathogenic mechanism underlying this multisystemic disorder. Multiple cell types, such as motor neurons and glial cells in the central nervous system (CNS) and Schwann cells and skeletal muscle in peripheral tissues, collectively orchestrate the onset and progression of the disease [6,7,8,9]. Moreover, defects in cellular structure, physiology, and metabolism interact and reinforce each other, making it difficult to pinpoint a primary pathogenic mechanism and develop effective therapeutics [6,7,8,9]. Current FDA-approved medications, such as the glutamate antagonist Riluzole, the free radical scavenger Edaravone, or the endoplasmic reticulum stress and mitochondrial dysfunction mitigator Relyvrio, only extend patients’ lives for a few months, with no capacity to reverse nerve damage or muscle atrophy [10,11,12].

**Figure 1:**
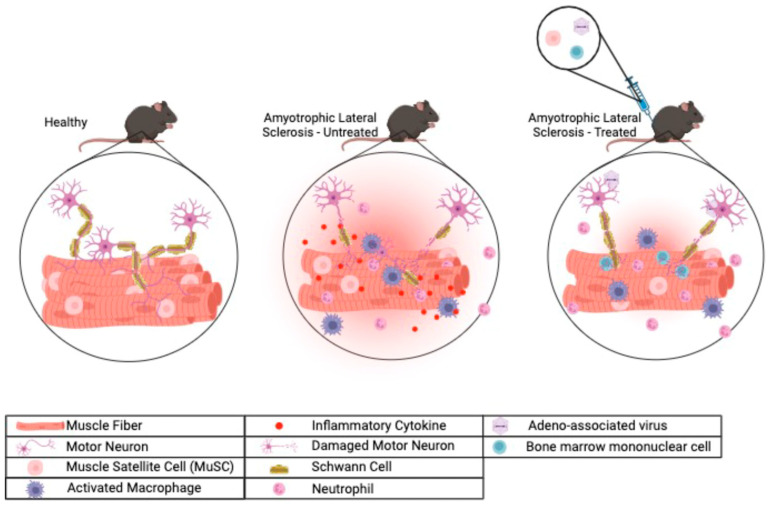
Dynamic interplay of skeletal muscle and nerves in response to therapeutics targeting skeletal muscles in ALS. In a healthy state, motor neurons are wrapped with Schwann cells to form the myelin sheath, aiding in neural signaling towards the NMJ and ultimately reaching the muscle fibers. In ALS, significant damage is evident in muscle fibers and Schwann cells, often accompanied by the presence of activated macrophages and neutrophils, releasing inflammatory cytokines within the skeletal muscle and the surrounding NMJ. This inflammatory milieu may signal back to the central nervous system and aggravate motor neuron degeneration. In response to therapeutics targeting skeletal muscle in ALS, such as stem cell transplantation or adeno-associated virus (AAV)-based gene therapies, a profound reduction in muscle inflammation is observed, which is paralleled by muscle regeneration and NMJ recovery, leading to neuroprotection and regeneration.

In ALS, muscle atrophy has traditionally been viewed as a secondary consequence of MN degeneration and denervation [13]. However, distinct pathologies within the skeletal muscle, such as fiber necrosis, inflammation, and myopathic features, have been documented in ALS muscles [14,15,16], suggesting that skeletal muscle atrophy in ALS may not solely result from denervation. Furthermore, studies of ALS patients have indicated that skeletal muscle plays an early and active role in the development of ALS [17,18]. Defects in skeletal muscle and the neuromuscular junction (NMJ) can occur before MN degeneration and the onset of clinical symptoms in ALS animal models and patients [19,20,21]. Skeletal muscle-restricted expression of an ALS-associated mutated protein (a G93A mutant form of human superoxide dismutase type 1 (SOD1)) causes motor neuron degeneration and a fatal ALS-like syndrome in transgenic mice [22,23,24,25]. All these observations suggest that ALS can be a distal axonopathy. These findings have brought renewed attention to the ‘dying-back’ hypothesis for ALS, where a retrograde pathogenic signaling cascade that originates in the peripheral tissues, such as skeletal muscles, can trigger the degeneration of MNs in the CNS [17,18]. Intriguingly, evidence of distal axonopathy has also surfaced in other neurodegenerative diseases, such as Parkinson’s disease and Alzheimer’s disease, suggesting that targeting peripheral tissues may be an effective therapeutic strategy for a range of neurodegenerative disorders, including ALS [26,27,28]. This review aims to provide a comprehensive summary of studies on therapies that target skeletal muscle and their impact on muscle satellite cells (MuSCs), inflammation, and the NMJ, which are essential components of ALS pathogenesis (summarized in Figure 1 and Table 1).

## 2. Muscle Satellite Cells

Skeletal muscle regeneration is a well-coordinated process of myogenesis that relies on MuSCs activation, proliferation, fusion, and differentiation [29]. Quiescent MuSCs are activated in response to acute injury, muscle denervation, or exercise stimuli. Once activated, MuSCs enter the cell cycle for proliferation, followed by differentiation into myoblasts and fusion with existing myofibers to repair and regenerate muscle fibers. The orderly process of myogenesis is tightly regulated by a family of transcription factors known as the myogenic regulatory factors (MRFs). They are crucial for regulating the gene expression essential for specifying the skeletal muscle lineage and controlling myogenic differentiation [30]. MuSCs express specific MRFs depending on their state. In their quiescent state, they express the MRF family member *Pax7* (a paired box family transcription factor) and myogenic factor 5 (*Myf5*) [31,32]. Upon activation, MuSCs quickly enter the cell cycle and begin to express another MRF member, *MyoD* (myogenic differentiation 1) [33]. The co-expression of both *Pax7* and *MyoD* indicates that these stem cells have shifted to an active state. As activated MuSCs proliferate, *Pax7* expression is downregulated, while *MyoD* and/or *Myf5* expression persists. During differentiation, another MRF, myogenin (*MyoG*; *Myf4*), is expressed and is essential for the differentiation of MuSCs into multinucleated myotubes, making myogenin a marker for the onset of myogenic differentiation [33]. Besides muscle regeneration, MuSCs also help maintain the structure and activity of the NMJ [34,35]. Depletion of MuSCs results in impaired myofiber/NMJ connectivity and inefficient re-innervation of the NMJ [34]. 

In ALS, the activity and function of MuSCs are altered. Pradat and colleagues isolated MuSCs from the deltoid muscle biopsies of ALS patients and observed that these cells proliferated at a similar rate to those from healthy controls [36]. However, the myoblasts derived from ALS patients displayed a senescent-like morphology, with increased senescence markers, including senescent-associated (SA)-βGal and p16 expression. In addition, MuSCs derived from ALS patients were unable to fully differentiate in vitro, which was evidenced by the abnormal morphology of the myotubes and reduced expression of MHC isoforms [36]. In another study, Scaramozza and colleagues observed that myoblasts derived from the vastus lateralis muscle of ALS patients exhibited a higher proliferation rate than those of control cultures [37]. Additionally, these ALS-derived myoblasts displayed significantly higher transcription levels of *MyoD* compared to controls, while maintaining similar levels of *Pax7*. Ultrastructural assays revealed that ALS myoblasts had an altered morphology characterized by a large number of vacuoles. Furthermore, differentiating ALS myoblasts displayed lower expression levels of *Myf4* compared to controls [37]. Studies by both Pradat and Scaramozza suggest that myoblasts derived from ALS patients are unable to fully differentiate into myotubes to achieve efficient muscle regeneration. Mitochondrial bioenergetics failure was observed in satellite cells isolated from the early presymptomatic stage (p55) of an established ALS mouse model carrying human mutated SOD1 (G93A) genes [38]. Dysfunctional mitochondria accelerate the accumulation of reactive oxygen species and other DNA-damaging factors. These factors may contribute to the susceptibility of MuSCs to degeneration following the activation of the repair system [37]. Although further research is necessary to elucidate the mechanisms underlying the compromised muscle regeneration mediated by MuSCs in ALS, these cells could represent a potential therapeutic target for the disease.

### Therapeutic Targets on MuSCs

Recently, researchers have explored the purinergic P2X receptor 7 (P2XR7), a family member of purinergic ionotropic receptors, as a therapeutic target for ALS, aiming to promote muscle regeneration. For example, Fabbrizio and colleagues found that activation of P2XR7 was able to reduce muscle atrophy in ALS mice [39]. Activation of P2XR7 through intramuscular administration of the P2XR7 agonist 2′(3′)-O-(4-benzoylbenzoyl) adenosine 5-triphosphate (BzATP) into the tibialis anterior (TA), gastrocnemius medialis (GCM), and quadriceps (QC) of SOD1 (G93A) mice enhances the pro-regenerative activity of infiltrating macrophages and improves the motor performance of ALS mice by promoting the activation and differentiation of MuSCs [40]. BzATP-treated muscle exhibited a larger muscle fiber cross-sectional area and higher expression levels of myogenic factors (*MyoD* and *MyoG*) in the QC muscle compared to controls. Primary satellite cells isolated from BzATP-treated mice also showed increased proliferation rates and fusion index during differentiation. A serial of in vitro evaluations confirmed that BzATP promotes satellite cell proliferation and differentiation, which were mediated by the activation of P2XR7. Additionally, supplements of BzATP resulted in decreased glycogen synthase kinase 3 (GSK3) activation and increased extracellular signal-regulated kinase 1/2 (ERK1/2) phosphorylation in the skeletal muscle of SOD1 (G93A) mice, suggesting the involvement of pro-survival/regenerative pathways. In addition, activation of P2XR7 by BzATP increased the recruitment of CD11b^+^ cells (macrophages) in the skeletal muscles of SOD1 (G93A) mice, particularly at the disease onset stage. Histological analysis revealed an increase in Macrophage 2 (M2) CD206^+^ macrophages in BzATP-treated mice, while Macrophage 1 (M1) iNOS^+^ macrophages showed no significant difference compared to controls, suggesting a correlation between P2XR7 activation and M2 polarization. At the onset of the disease, BzATP treatment led to downregulation of pro-inflammatory cytokines like insulin-like growth factor-1 (IGF-1) and Tumor Necrosis Factor α (TNF-α) while increasing the anti-inflammatory cytokine interleukin 10 (IL-10), indicating a shift towards an anti-inflammatory milieu favoring muscle regeneration [39]. Other studies have shown that activating P2X7 benefits the peripheral nervous system (PNS) by promoting Schwann cell proliferation after sciatic nerve injury and facilitating myogenesis and the formation of the NMJ [39,41]. 

In addition to P2XR7, mouse insulin-like growth factor (mlgf)-1 isoform, previously implicated in the anabolism of muscle and nerve tissues, has shown promising potential as a muscle-focused ALS treatment [42]. Dobrowolny and colleagues found that the skeletal muscle-restricted expression of mlgf-1 enhanced MuSC activity and fiber maturation [42]. Transgenic mIgf-1 expression also stabilized the NMJ, reduced spinal cord inflammation, improved MN survival, and ultimately prolonged the lifespan of mice carrying the ALS-associated SOD1 (G93A) mutation [42]. These studies highlight the potential of targeting MuSCs for ALS therapies. 

## 3. Inflammation

Neuroinflammation has been recognized as one of the key mediators of ALS pathogenesis. Zamiri and colleagues identified immunological dysregulation as a central contributor to disease progression in sporadic ALS, making it a potential therapeutic target [43]. They observed infiltration of immune cells such as IL-17A and granzyme-positive cytotoxic T lymphocytes (CTLs), IL-17A-positive mast cells, and inflammatory macrophages into the brain and spinal cord. Early elevation in inflammatory cytokines (IL-12A, IFN-γ, TNF-α), granzymes, and transcription factors (*STAT3*, *STAT4*) in peripheral blood mononuclear cells (PBMCs) was also observed. Upregulation of autoimmunity-associated cytokines (*IL-23A*, *IL-17B*) and chemokines (*CXCL9*, *CXCL10*) in PBMCs was detected, attracting CTLs and monocytes into the CNS. Furthermore, systemic inflammation in ALS is also driven by changes in T-cell regulation. In ALS patients, inhibitory co-receptors like CTLA4 (cytotoxic T lymphocyte-associated protein 4) and PD-1 (programmed cell death protein-1) decrease, while stimulatory co-receptors like *OX40* and *GITR* increase. CTLA4 gradually decreases over time, while LAG3 initially increases but sharply declines around 40 months post-onset. Conversely, *OX40* and *GITR* significantly upregulate in the same time frame. Longer-surviving patients exhibit increased FOXP3 activity, a key regulator of regulatory T cells (Tregs), aiding immune response regulation. In addition, proteomic analysis revealed heightened expression of granzymes, kinases, cell adhesion, and apoptotic proteins in the natural killer (NK) cells of an ALS patient compared to their healthy twin. Investigations into therapeutic interventions for sporadic ALS patients revealed that dimethyl fumarate (DMF) and the cGAS/STING pathway inhibitor H-151 downregulate granzymes and pro-inflammatory cytokines (IL-1β, IL-6, IL-15, IL-23A, IFN-γ), promoting a pro-resolution macrophage phenotype. Additionally, anti-inflammatory eicosanoid epoxyeicosatrienoic acids (EETs) from arachidonic acid synergize with DMF. DMF and H-151 emerge as promising drugs that target inflammation and autoimmunity in sporadic ALS by modulating the NFκB and cGAS/STING pathways [43].

Dysregulated inflammatory processes in the skeletal muscle also play a significant role in ALS pathology [15]. For healthy muscles, when muscle injury occurs, resident and recruited mast cells, neutrophils, and other immune cells contribute to creating a pro-inflammatory environment through the secretion of pro-inflammatory cytokines, such as TNF-α, interferon-γ (IFN-γ), and interleukin-1β (IL-1β). This process recruits macrophages, derived from monocytes in the bone marrow, to the site of muscle injury at approximately two days post-injury. During muscle regeneration, the macrophages undergo a transition from the M1 (pro-inflammatory) to the M2 (anti-inflammatory) phase. In the early stage of muscle regeneration, M1 macrophages predominate, promoting the proliferation of MuSCs by secreting large amounts of cytokines such as TNF-α, IL-1β, IGF-1, interleukin-6 (IL-6), and IFN-γ [44]. As the number of MuSCs reaches its peak, the pro-inflammatory (M1) microenvironment transitions into the anti-inflammatory state (M2), facilitating the differentiation of MuSCs and the maturation of newly formed myofibers. M2 macrophages establish this anti-inflammatory environment by producing anti-inflammatory cytokines, such as interleukin-4 (IL-4), interleukin-10 (IL-10), and interleukin-13 (IL-13), and suppressing the local inflammatory response at the injury site. Simultaneously, M2 macrophages facilitate the differentiation of MuSCs into myotubes, thereby contributing to the later stages of myogenesis and regeneration through secreting various growth factors, such as GDF3 [45,46]. This transition from the pro-inflammatory to the anti-inflammatory macrophage phenotype during muscle regeneration is crucial to maintaining a favorable regeneration microenvironment.

In the ALS in vivo model, inflammation gradually worsens as the disease advances from the late pre-symptomatic stage to the symptomatic and late disease states in the limb muscles. This inflammation is particularly prominent near the postsynaptic region of the NMJ [47]. Elevated inflammasome activation has been implicated in skeletal muscle pathology in ALS, as evidenced by macrophage infiltration and increased levels of caspase-1 and IL-1β in both the SOD1 (G93A) mouse model and sporadic ALS patients [48]. Furthermore, the elevated levels of these proteins in the skeletal muscle of pre-symptomatic SOD1 (G93A) mice indicate an early activation of innate immunity in the pathogenesis of ALS [48]. In addition, during muscle regeneration, elevated IL-1β levels correlate with an accumulation of activated macrophages, leading to impaired regeneration [49]. Prolonged inflammation disrupts the microenvironment of cells within the skeletal muscle, upsets the delicate equilibrium between protein synthesis and degeneration, and affects components like the myosin heavy chain, the major contractile protein required to sustain muscle contraction [50]. This disruption is achieved through the activation of various inflammatory signaling pathways, including but not limited to the NF-κB (nuclear factor-κB), JAK/STAT (Janus-activated kinase/signal transducer and activator of transcription), and p38 MAPK (mitogen-activated protein kinase) pathways [51]. Therefore, modulating inflammation in ALS skeletal muscle may facilitate tissue regeneration and ameliorate disease progression.

### Therapeutic Targets for Anti-Inflammation

During muscle regeneration, macrophages are the dominant immune cells recruited within the damaged tissue and directly interplay with MuSCs to orchestrate their fate through different secretory cues [45]. Intramuscular injection of the anti-inflammatory factor IL-10 into SOD1 (G93A) mice has been shown to counteract skeletal muscle atrophy by facilitating macrophage polarization and MuSCs differentiation [52]. Upon IL-10 administration, protein levels of MyoD and MyoG were significantly increased in the MuSCs, indicating a promotive role in facilitating the transition from the proliferative stage to the differentiation stage [53]. IL-10, as a potent immunomodulatory factor, can induce the shift of M1 macrophages to M2, which is crucial for muscle growth and regeneration. The study found that IL-10 treatment increased CD11b^+^ cell density in the TA muscle, suggesting a potential influence on macrophage proliferation. In vitro, SOD1 (G93A) macrophages showed a 2.7-fold increase in proliferation with IL-10 treatment, which was reversed upon IL-10 blockage. In C57-SOD1 (G93A) mice, IL-10 reduced M1 iNOS^+^ macrophages while increasing their M2 CD206^+^ counterparts in the quadriceps muscle. Histological and RNA lysate analyses confirmed the decreased expression of pro-inflammatory cytokines (TNFα and IL1-β) with IL-10 treatment, indicating an anti-inflammatory effect. The study further investigated the influence of IL-10 on the interaction between macrophages and MuSCs in damaged skeletal muscle. The administration of IL-10 enhanced the migration of macrophages towards MuSCs in vitro, even in the absence of M2 polarization. This effect was further boosted when IL-10 was combined with IL-4, indicating enhanced macrophage–MuSCs crosstalk. Eventually, the immunomodulation and anti-inflammation mediated by IL-10 led to the preservation of MNs, improving motor performance and extending the lifespan of SOD1 (G93A) mice [52]. 

Trolese and colleagues also demonstrated that boosting the peripheral immune response by utilizing an intramuscular injection of the scAAV9 vector packed with *Mcp1* (monocyte chemoattractant protein-1) improved motor functions in SOD1 (G93A) mice [54]. *Mcp1* is a key chemokine that regulates the migration and infiltration of monocytes/macrophages. This intervention, in turn, triggered the differentiation of myogenic progenitors and facilitated muscle re-innervation, ultimately leading to improved muscle strength and a delay in disease onset. The study also revealed that the fluorescent protein-tagged scAAV9 vector spread retrogradely from the injected muscles alongside the motor unit, eventually transducing the soma of MNs [54]. The induction of *Mcp1* in the motor unit protected MNs in the spinal cord by decreasing neuroinflammation, as indicated by decreased pro-inflammatory markers, such as IL-1β [54]. Together, these studies suggest that the direct modulation of inflammation in skeletal muscle not only stimulates muscle regeneration but also preserves the NMJ and enhances motor function in an ALS mouse model.

## 4. Neuromuscular Junction

Given the early pathological changes that occur at the NMJ prior to the onset of ALS clinical symptoms, the NMJ is emerging as a promising therapeutic target for ALS treatment. The NMJ directly links the nervous and muscular systems and enables communication between the MNs and skeletal muscle fibers [55]. It comprises three essential elements: the presynaptic motor nerve terminal, the perisynaptic Schwann cells (SCs), and the postsynaptic plasma membrane of the muscle fiber [56,57]. Upon the arrival of the action potential, calcium enters the presynaptic terminal, promoting the release of the neurotransmitter acetylcholine (ACh) into the extracellular space. The ACh then binds to tightly clustered ACh receptors (AChRs) on the muscle tissue, initiating the muscle action potential that ultimately leads to muscle contraction [58]. The malfunction of the NMJ disrupts muscle contraction, highlighting its central role in neuromuscular disorders [58]. In support of the distal axonopathy theory of ALS, pathological changes in the NMJ were reported to occur before MN degeneration and the onset of clinical symptoms [7,59,60]. Studies have revealed deficiencies in NMJ formation, fidelity, stability, and fatigability across all ALS NMJ models [61]. Additionally, there is a significant decrease in the innervated endplate area and increased fragmentation of the NMJ in ALS [61]. 

### Therapeutic Targets for the NMJ

To ameliorate NMJ and MN defects in ALS patients, Barrientos and colleagues injected bone marrow mononuclear cells (BMMCs) into the skeletal muscle in a phase I/II clinical trial [62]. The results revealed a significant increase in the compound muscle action potential (CMAP), which was indicated by the D50 index, within the treatment group, suggesting that BMMCs have the potential to ameliorate NMJ damage in ALS [62]. In another study, human umbilical cord blood-derived mesenchymal stem cells (hUCB-MSCs) were repeatedly intramuscularly injected into the limbs of SOD1 (G93A) mice [63]. The treated group exhibited a significant enlargement of the NMJ endplate area compared to the vehicle-treated group. Implanted hUCB-MSCs also ameliorated muscle atrophy by inhibiting reactive oxidative species generation and activating AMPK-regulated protein synthesis. As a result, the transplantation of hUCB-MSCs improved motor function and extended survival in an ALS mouse model [63]. In addition, there are other treatments that combine stem cells with neurotrophic factors, such as glial-derived neurotrophic factors (GDNFs) and vascular endothelial growth factor (VEGF), to target the NMJ and muscle atrophy in an ALS animal model [64,65]. GDNFs and VEGF have also been shown to protect motor neurons in ALS models [66,67]. Intramuscular injection of human mesenchymal stem cells (hMSCs) engineered to express GDNF and/or VEGF led to a significant improvement in innervated endplates, as indicated by increased AChR cluster formation in an ALS animal model [64]. This protective effect extended to MNs in the spinal cord, resulting in an overall increase in the survival of SOD1 (G93A) rats [64]. In another study, overexpression of neuregulin (NRG-1), a neurotrophic factor that supports axonal and neuromuscular development and maintenance, within the skeletal muscle contributed to NMJ maintenance and improved redox homeostasis in the muscle of SOD1 (G93A) mice [68,69,70,71]. This also led to decreased glial reactivity and enhanced MN survival in the spinal cord [71]. Therefore, neurotrophic factors in conjunction with stem cells may serve as a valuable therapeutic target for ALS, as demonstrated by their ability to ameliorate both NMJ and MN phenotypes.

In addition, gene therapy for ALS has also been successfully administered in an ALS mouse model by intramuscular injection of AAV to express human Dok-7, a crucial muscle protein involved in NMJ formation [72]. This treatment effectively reduced muscle denervation and improved motor function [72]. Nonetheless, the perceived safety of the direct administration of gene editing-based therapy into humans warrants further studies to ensure that there are no short- and long-term side effects. Overall, these studies show promising results for targeting the NMJ as a treatment for ALS.

**Table 1:** Therapeutics targeting skeletal muscles for ALS treatment.

Drug/Approach	Animal Model	Motor Performance	Effects on MNs in Spinal Cord	Effects on NMJ	Effects on Inflammation	Effects on Muscle Satellite Cells	Effects on Disease Onset	Lifespan	Reference
2′-(3′)-O-(4-benzoyl-benzoyl) adenosine 5′-triphosphate triethylammonium salt (BzATP)	B6.Cg-Tg(mSOD1)1Gur/J mouse	Delayed onset of motor impairment			Reduced inflammation in the spinal cord	Increased activation and differentiation	Delayed onset of motor impairment		[40]
Restricted skeletal muscle expression of insulin-like growth factor (mIgf)-1 isoform	SOD1G93A B6J mice		Enhanced MN survival	Stabilized NMJ	Reduced inflammation in the spinal cord	Increased activation and differentiation	Delayed onset of motor impairment	Extended lifespan	[42]
Intramuscular administration of recombinant mouse IL-10	SOD1G93A male mice and C57BL/6J (C57-SOD1G93A) and 129S2/Sv (129Sv-SOD1G93A) female mice	Delayed onset of motor impairment	Enhanced MN survival		Reduced inflammation in the spinal cord and enhanced macrophage polarization to Macrophage 2 in skeletal muscle	Increased activation and differentiation	Delayed onset and progression of muscle strength impairment	Extended lifespan	[52]
Intramuscular administration of scAAV9 vector engineered with the Mcp1 gene	SOD1G93A mice on C57BL/6J or 129SvHsd backgrounds	Delayed onset of motor impairment	Enhanced MN survival		Enhanced expression of anti-inflammatory markers	Increased activation and differentiation	Delayed onset and progression of muscle strength impairment		[54]
Intramuscular transplantation of human umbilical cord blood-derived mesenchymal stem cells (hUCB-MSCs)	hSOD1G93A transgenic mice (B6.Cg-Tg(SOD1*G93A)1Gur/J)	Improved motor function and activity		Reduced NMJ degeneration		Decreased muscle atrophy	Delayed onset of disease indicated by body weight loss	Extended lifespan	[63]
Human mesenchymal stem cells engineered to secrete glial cell line-derived neurotrophic factor (hMSC-GDNF)	SOD1G93A rats		Enhanced MN survival	Reduced NMJ denervation			No significant difference in disease onset or progression	Extended lifespan	[64]
Human mesenchymal stem cells engineered to secrete glial cell line-derived neurotrophic factor (hMSC-GDNF) and vascular endothelial growth factor (hMSC-VEGF)	SOD1G93A rats	Delayed onset of motor impairment	Enhanced MN survival	Reduced NMJ denervation			Delayed disease onset	Extended lifespan	[65]
Vascular endothelial growth factor (VEGF)-expressing lentiviral vector	SOD1G93A mice	Decreased motor impairment	Enhanced MN survival				Delayed disease onset and progression	Extended lifespan	[67]
Skeletal muscle overexpression of Neuregulin 1 type I (NRG1-I)	SOD1G93A mice	Improved motor function and activity	Enhanced MN survival	Reduced NMJ denervation	Reduced neuron inflammation		Delayed disease onset		[71]

## 5. Mitochondria

Mitochondria, which support various essential cellular processes, including energy production, calcium storage, and lipid synthesis, are vital for cell viability and maintenance of life. In ALS patients, both sporadic and familial cases exhibit similar mitochondrial abnormalities in the spinal cord and muscles, as characterized by defects in morphology, quantity, and disposition. These abnormalities are accompanied by defects in the respiratory chain complex and increased oxidative stress [73]. Research by Bernardini et al. (2013) utilizing microarray technology identified significant alterations in mitochondrial network gene expressions in ALS, which are crucial for oxidative phosphorylation and ATP synthesis in muscle tissues [74]. Further studies using multigene qRT-PCR revealed a downregulation of genes responsible for mitochondrial biogenesis and dynamics, indicating a pervasive mitochondrial dysfunction in ALS [75]. Defective mitochondrial respiratory function, a primary source of reactive oxygen species (ROS) production, can lead to elevated intracellular ROS levels. Alleviated ROS and the resulting oxidative stress play a role in the pathogenesis of ALS, potentially leading to the formation of the unfolded protein aggregates that are invariably found in ALS motor neurons [76]. Halter B observed that the accumulation of ROS in skeletal muscles occurs at the asymptomatic stage in SOD1 (G93A) mice [77]. Similarly, Méndez-López I and Scaricamazza S found that mitochondrial dysfunction in the skeletal muscles of SOD1 (G93A) mice occurs before clinical symptoms appear [38,78]. These studies suggest that mitochondria and the resulting oxidative stress may contribute to the onset and progression of ALS. 

### Therapeutic Targets for Mitochondrial Dysfunction and Oxidative Stress

Edaravone (MCI-186, 3-methyl-1 phenyl-2-pyrazolin-5-one), first described as a free radical scavenger, has been approved for treating ALS since 2015 in several countries, including Japan, South Korea, the United States, and Canada [79]. Edaravone treatment of rats with cerebral infarction significantly boosts the expression of Nrf2 (nuclear factor erythroid 2-related factor-2), a key stimulator of antioxidant activities that defend against oxidative stress [80]. Nrf2 activation, triggered by inflammation or injury, leads to its translocation from the cytoplasm to the nucleus, where it binds to antioxidant response elements in the promoter regions of various detoxifying enzymes, such as HO-1 (heme oxygenase-1) and NQO1 (NAD(P)H quinone oxidoreductase-1), thereby protecting cells from oxidative damage [81]. Additionally, Honokiol has demonstrated the ability to reduce cellular oxidative stress by enhancing the synthesis of glutathione (GSH) and activating the Nrf2 antioxidant response element (ARE) pathway. It also improves mitochondrial efficiency and morphology, promoting mitochondrial dynamics in SOD1(G93A) cells. Notably, Honokiol extends the lifespan and enhances the motor function of SOD1(G93A) transgenic mice when administered daily from disease onset to the end stage [82]. ALCAT1 (acyl-CoA:lysocardiolipin acyltransferase 1), an acyltransferase associated with mitochondrial dysfunction in age-related diseases, has been found to have increased expression in the skeletal muscle of SOD1(G93A) mice. Targeted deletion of ALCAT1 and pharmacological inhibition of this enzyme can prevent the aggregation of SOD1 (G93A) protein and mitochondrial dysfunction. These interventions may help to attenuate motor neuron dysfunction, skeletal muscle atrophy, and neuronal inflammation in SOD1 (G93A) mice [83]. Sodium butyrate has been shown to improve mitochondrial respiration and alleviate disease progression in ALS models [84,85,86]. Additionally, it promotes MuSC renewability and increases the expression of *Cxcl12*, which aids in axon attraction. Supplementation with sodium butyrate also resulted in reduced NMJ loss in the hindlimb and diaphragm muscles of SOD1 (G93A) mice [87]. These studies suggest that alleviating mitochondrial dysfunction and oxidative stress could significantly improve disease symptoms and influence the progression of ALS in cellular and animal models. 

## 6. Conclusions

As a multisystem disorder, ALS underscores the critical yet often overlooked involvement of skeletal muscle in the onset and progression of the disease. The dysregulation of MuSC activation and differentiation, chronic inflammation, and dismantling of the NMJ collectively shape retrograde signaling that may contribute significantly to MN degeneration and exacerbate ALS syndromes. These skeletal muscle-related defects represent therapeutic targets with the potential to delay or even reverse the progression of the disease. This review has summarized therapies targeting MuSCs, inflammation, and the NMJ within the skeletal muscle as potential treatments for ALS. These therapies have exhibited numerous beneficial effects, including the reduction in inflammation and mitochondria defects and the amelioration of muscle and motor functions. These improvements led to extended lifespan in ALS in vivo models, supporting the critical roles of skeletal muscles in understanding and treating ALS. These findings further demonstrate the intricate interplay between multiple tissues and cell types in the complexity of ALS, which will aid in identifying novel pathogenic mechanisms and innovative therapeutic targets for the treatment of ALS.

## References

[1] Byrne S., Walsh C., Lynch C., Bede P., Elamin M., Kenna K., McLaughlin R., Hardiman O. (2011). Rate of familial amyotrophic lateral sclerosis: A systematic review and meta-analysis. J. Neurol. Neurosurg. Psychiatry.

[2] Zufiría M., Gil-Bea F.J., Fernández-Torrón R., Poza J.J., Muñoz-Blanco J.L., Rojas-García R., Riancho J., de Munain A.L. (2016). ALS: A bucket of genes, environment, metabolism and unknown ingredients. Prog. Neurobiol..

[3] Mathis S., Goizet C., Soulages A., Vallat J.M., Le Masson G. (2019). Genetics of amyotrophic lateral sclerosis: A review. J. Neurol. Sci..

[4] Cleveland D.W., Rothstein J.D. (2001). From Charcot to Lou Gehrig: Deciphering selective motor neuron death in ALS. Nat. Rev. Neurosci..

[5] Andersen P.M., Al-Chalabi A. (2011). Clinical genetics of amyotrophic lateral sclerosis: What do we really know?. Nat. Rev. Neurol..

[6] Philips T., Rothstein J.D. (2014). Glial cells in amyotrophic lateral sclerosis. Exp. Neurol..

[7] Moloney E.B., de Winter F., Verhaagen J. (2014). ALS as a distal axonopathy: Molecular mechanisms affecting neuromuscular junction stability in the presymptomatic stages of the disease. Front. Neurosci..

[8] Phatnani H.P., Guarnieri P., Friedman B.A., Carrasco M.A., Muratet M., O’Keeffe S., Nwakeze C., Pauli-Behn F., Newberry K.M., Meadows S.K. (2013). Intricate interplay between astrocytes and motor neurons in ALS. Proc. Natl. Acad. Sci. USA.

[9] Shefner J.M., Musaro A., Ngo S.T., Lunetta C., Steyn F.J., Robitaille R., De Carvalho M., Rutkove S., Ludolph A.C., Dupuis L. (2023). Skeletal muscle in amyotrophic lateral sclerosis. Brain.

[10] Hinchcliffe M., Smith A. (2017). Riluzole: Real-world evidence supports significant extension of median survival times in patients with amyotrophic lateral sclerosis. Degener. Neurol. Neuromuscul. Dis..

[11] Cho H., Shukla S. (2021). Role of Edaravone as a Treatment Option for Patients with Amyotrophic Lateral Sclerosis. Pharmaceuticals.

[12] Paganoni S., Macklin E.A., Hendrix S., Berry J.D., Elliott M.A., Maiser S., Karam C., Caress J.B., Owegi M.A., Quick A. (2020). Trial of Sodium Phenylbutyrate-Taurursodiol for Amyotrophic Lateral Sclerosis. N. Engl. J. Med..

[13] Veltema A.N. (1975). The case of the saltimbanque Prosper Lecomte. A contribution to the study of the history of progressive muscular atrophy (Aran-Duchenne) and amyotrophic lateral sclerosis (Charcot). Clin. Neurol. Neurosurg..

[14] Iwasaki Y., Sugimoto H., Ikeda K., Takamiya K., Shiojima T., Kinoshita M. (1991). Muscle morphometry in amyotrophic lateral sclerosis. Int. J. Neurosci..

[15] Al-Sarraj S., King A., Cleveland M., Pradat P.F., Corse A., Rothstein J.D., Leigh P.N., Abila B., Bates S., Wurthner J. (2014). Mitochondrial abnormalities and low grade inflammation are present in the skeletal muscle of a minority of patients with amyotrophic lateral sclerosis; an observational myopathology study. Acta Neuropathol. Commun..

[16] Amrit A.N., Anderson M.S. (1974). Serum creatine phosphokinase in amyotrophic lateral sclerosis. Correlation with sex, duration, and skeletal muscle biopsy. Neurology.

[17] Schiffman P.L., Belsh J.M. (1993). Pulmonary-Function at Diagnosis of Amyotrophic-Lateral-Sclerosis—Rate of Deterioration. Chest.

[18] Tsitkanou S., Lindsay A., Della Gatta P. (2019). The role of skeletal muscle in amyotrophic lateral sclerosis: A ‘dying-back’ or ‘dying-forward’ phenomenon?. J. Physiol..

[19] Coleman M. (2005). Axon degeneration mechanisms: Commonality amid diversity. Nat. Rev. Neurosci..

[20] Frey D., Schneider C., Xu L., Borg J., Spooren W., Caroni P. (2000). Early and selective loss of neuromuscular synapse subtypes with low sprouting competence in motoneuron diseases. J. Neurosci..

[21] Dadon-Nachum M., Melamed E., Offen D. (2011). The “Dying-Back” Phenomenon of Motor Neurons in ALS. J. Mol. Neurosci..

[22] Lino M.M., Schneider C., Caroni P. (2002). Accumulation of SOD1 mutants in postnatal motoneurons does not cause motoneuron pathology or motoneuron disease. J. Neurosci..

[23] Martin L.J., Wong M. (2020). Skeletal Muscle-Restricted Expression of Human SOD1 in Transgenic Mice Causes a Fatal ALS-Like Syndrome. Front. Neurol..

[24] Wong M., Martin L.J. (2010). Skeletal muscle-restricted expression of human SOD1 causes motor neuron degeneration in transgenic mice. Hum. Mol. Genet..

[25] Dobrowolny G., Aucello M., Musaro A. (2011). Muscle atrophy induced by SOD1G93A expression does not involve the activation of caspase in the absence of denervation. Skelet. Muscle.

[26] Arendt T. (2009). Synaptic degeneration in Alzheimer’s disease. Acta Neuropathol..

[27] Dauer W., Przedborski S. (2003). Parkinson’s disease: Mechanisms and models. Neuron.

[28] Selkoe D.J. (2002). Alzheimer’s disease is a synaptic failure. Science.

[29] Mauro A. (1961). Satellite cell of skeletal muscle fibers. J. Biophys. Biochem. Cytol..

[30] Zammit P.S. (2017). Function of the myogenic regulatory factors Myf5, MyoD, Myogenin and MRF4 in skeletal muscle, satellite cells and regenerative myogenesis. Semin. Cell Dev. Biol..

[31] Seale P., Sabourin L.A., Girgis-Gabardo A., Mansouri A., Gruss P., Rudnicki M.A. (2000). Pax7 is required for the specification of myogenic satellite cells. Cell.

[32] Tajbakhsh S., Rocancourt D., Cossu G., Buckingham M. (1997). Redefining the genetic hierarchies controlling skeletal myogenesis: Pax-3 and Myf-5 act upstream of MyoD. Cell.

[33] Fuchtbauer E.M., Westphal H. (1992). Myod and Myogenin Are Coexpressed in Regenerating Skeletal-Muscle of the Mouse. Dev. Dynam.

[34] Liu W., Wei-LaPierre L., Klose A., Dirksen R.T., Chakkalakal J.V. (2015). Inducible depletion of adult skeletal muscle stem cells impairs the regeneration of neuromuscular junctions. eLife.

[35] Liu W.X., Klose A., Forman S., Paris N.D., Pierre L.W.L., Cortés-Lopéz M., Tan A., Flaherty M., Miura P., Dirksen R.T. (2017). Loss of adult skeletal muscle stem cells drives age-related neuromuscular junction degeneration. eLife.

[36] Pradat P.F., Barani A., Wanschitz J., Dubourg O., Lombes A., Bigot A., Mouly V., Bruneteau G., Salachas F., Lenglet T. (2011). Abnormalities of satellite cells function in amyotrophic lateral sclerosis. Amyotroph. Lateral Scler..

[37] Scaramozza A., Marchese V., Papa V., Salaroli R., Soraru G., Angelini C., Cenacchi G. (2014). Skeletal muscle satellite cells in amyotrophic lateral sclerosis. Ultrastruct. Pathol..

[38] Scaricamazza S., Salvatori I., Giacovazzo G., Loeffler J.P., René F., Rosina M., Quessada C., Proietti D., Heil C., Rossi S. (2020). Skeletal-Muscle Metabolic Reprogramming in ALS-SOD1 Mice Predates Disease Onset and Is A Promising Therapeutic Target. iScience.

[39] Fabbrizio P., Apolloni S., Bianchi A., Salvatori I., Valle C., Lanzuolo C., Bendotti C., Nardo G., Volonte C. (2020). P2X7 activation enhances skeletal muscle metabolism and regeneration in SOD1G93A mouse model of amyotrophic lateral sclerosis. Brain Pathol..

[40] Fabbrizio P., D’Agostino J., Margotta C., Mella G., Panini N., Pasetto L., Sammali E., Raggi F., Soraru G., Bonetto V. (2021). Contingent intramuscular boosting of P2XR7 axis improves motor function in transgenic ALS mice. Cell Mol. Life Sci..

[41] Song X.M., Xu X.H., Zhu J., Guo Z., Li J., He C., Burnstock G., Yuan H., Xiang Z. (2015). Up-regulation of P2X7 receptors mediating proliferation of Schwann cells after sciatic nerve injury. Purinergic Signal.

[42] Dobrowolny G., Giacinti C., Pelosi L., Nicoletti C., Winn N., Barberi L., Molinaro M., Rosenthal N., Musaro A. (2005). Muscle expression of a local Igf-1 isoform protects motor neurons in an ALS mouse model. J. Cell Biol..

[43] Zamiri K., Kesari S., Paul K., Hwang S.H., Hammock B., Kaczor-Urbanowicz K.E., Urbanowicz A., Gao L., Whitelegge J., Fiala M. (2023). Therapy of autoimmune inflammation in sporadic amyotrophic lateral sclerosis: Dimethyl fumarate and H-151 downregulate inflammatory cytokines in the cGAS-STING pathway. Faseb J..

[44] Chen B.D., Shan T.Z. (2019). The role of satellite and other functional cell types in muscle repair and regeneration. J. Muscle Res. Cell M..

[45] Yang W.J., Hu P. (2018). Skeletal muscle regeneration is modulated by inflammation. J. Orthop. Transl..

[46] Garabuczi É., Tarban N., Fige É., Patsalos A., Halász L., Szendi-Szatmári T., Sarang Z., Király R., Szondy Z. (2023). Nur77 and PPARγ regulate transcription and polarization in distinct subsets of M2-like reparative macrophages during regenerative inflammation. Front. Immunol..

[47] Van Dyke J.M., Smit-Oistad I.M., Macrander C., Krakora D., Meyer M.G., Suzuki M. (2016). Macrophage-mediated inflammation and glial response in the skeletal muscle of a rat model of familial amyotrophic lateral sclerosis (ALS). Exp. Neurol..

[48] Lehmann S., Esch E., Hartmann P., Goswami A., Nikolin S., Weis J., Beyer C., Johann S. (2018). Expression profile of pattern recognition receptors in skeletal muscle of SOD1 amyotrophic lateral sclerosis (ALS) mice and sporadic ALS patients. Neuropath. Appl. Neuro.

[49] Cohen T.V., Many G.M., Fleming B.D., Gnocchi V.F., Ghimbovschi S., Mosser D.M., Hoffman E.P., Partridge T.A. (2015). Upregulated IL-1β in dysferlin-deficient muscle attenuates regeneration by blunting the response to pro-inflammatory macrophages. Skelet. Muscle.

[50] Balagopal P., Ljungqvist O., Nair K.S. (1997). Skeletal muscle myosin heavy-chain synthesis rate in healthy humans. Am. J. Physiol. ndocrinol. Metab..

[51] Ji Y.A., Li M., Chang M.Y., Liu R.Q., Qiu J.Y., Wang K.X., Deng C.Y., Shen Y.T., Zhu J.W., Wang W. (2022). Inflammation: Roles in Skeletal Muscle Atrophy. Antioxidants.

[52] Fabbrizio P., Margotta C., D’Agostino J., Suanno G., Quetti L., Bendotti C., Nardo G. (2023). Intramuscular IL-10 Administration Enhances the Activity of Myogenic Precursor Cells and Improves Motor Function in ALS Mouse Model. Cells.

[53] Tidball J.G. (2017). Regulation of muscle growth and regeneration by the immune system. Nat. Rev. Immunol..

[54] Trolese M.C., Scarpa C., Melfi V., Fabbrizio P., Sironi F., Rossi M., Bendotti C., Nardo G. (2022). Boosting the peripheral immune response in the skeletal muscles improved motor function in ALS transgenic mice. Mol. Ther..

[55] Slater C.R. (2017). The Structure of Human Neuromuscular Junctions: Some Unanswered Molecular Questions. Int. J. Mol. Sci..

[56] Sanes J.R., Lichtman J.W. (1999). Development of the vertebrate neuromuscular junction. Annu. Rev. Neurosci..

[57] Court F.A., Gillingwater T.H., Melrose S., Sherman D.L., Greenshields K.N., Morton A.J., Harris J.B., Willison H.J., Ribchester R.R. (2008). Identity, developmental restriction and reactivity of extralaminar cells capping mammalian neuromuscular junctions. J. Cell Sci..

[58] Cruz P.M.R., Cossins J., Beeson D., Vincent A. (2020). The Neuromuscular Junction in Health and Disease: Molecular Mechanisms Governing Synaptic Formation and Homeostasis. Front. Mol. Neurosci..

[59] Fischer L.R., Culver D.G., Tennant P., Davis A.A., Wang M.S., Castellano-Sanchez A., Khan J., Polak M.A., Glass J.D. (2004). Amyotrophic lateral sclerosis is a distal axonopathy: Evidence in mice and man. Exp. Neurol..

[60] Jokic N., de Aguilar J.L.G., Dimou L., Lin S., Fergani A., Ruegg M.A., Schwab M.E., Dupuis L., Loeffler J.P. (2006). The neurite outgrowth inhibitor Nogo-A promotes denervation in an amyotrophic lateral sclerosis model. Embo Rep..

[61] Badu-Mensah A., Guo X.F., Nimbalkar S., Cai Y.Q., Hickman J.J. (2022). ALS mutations in both human skeletal muscle and motoneurons differentially affects neuromuscular junction integrity and function. Biomaterials.

[62] Geijo-Barrientos E., Pastore-Olmedo C., De Mingo P., Blanquer M., Espuch J.G., Iniesta F., Iniesta N.G., García-Hernández A., Martín-Estefanía C., Barrios L. (2020). Intramuscular Injection of Bone Marrow Stem Cells in Amyotrophic Lateral Sclerosis Patients: A Randomized Clinical Trial. Front. Neurosci..

[63] Kook M.G., Lee S., Shin N., Kong D., Kim D.H., Kim M.S., Kang H.K., Choi S.W., Kang K.S. (2020). Repeated intramuscular transplantations of hUCB-MSCs improves motor function and survival in the SOD1 G93A mice through activation of AMPK. Sci. Rep..

[64] Suzuki M., McHugh J., Tork C., Shelley B., Hayes A., Bellantuono I., Aebischer P., Svendsen C.N. (2008). Direct Muscle Delivery of GDNF with Human Mesenchymal Stem Cells Improves Motor Neuron Survival and Function in a Rat Model of Familial ALS. Mol. Ther..

[65] Krakora D., Mulcrone P., Meyer M., Lewis C., Bernau K., Gowing G., Zimprich C., Aebischer P., Svendsen C.N., Suzuki M. (2013). Synergistic Effects of GDNF and VEGF on Lifespan and Disease Progression in a Familial ALS Rat Model. Mol. Ther..

[66] Henderson C.E., Phillips H.S., Pollock R.A., Davies A.M., Lemeulle C., Armanini M., Simpson L.C., Moffet B., Vandlen R.A., Koliatsos V.E. (1994). Gdnf—A Potent Survival Factor for Motoneurons Present in Peripheral-Nerve and Muscle. Science.

[67] Azzouz M., Ralph G.S., Storkebaum E., Walmsley L.E., Mitrophanous K.A., Kingsman S.M., Carmeliet P., Mazarakis N.D. (2004). VEGF delivery with retrogradely transported lentivector prolongs survival in a mouse ALS model. Nature.

[68] Syroid D.E., Maycox P.R., Burrola P.G., Liu N.L., Wen D.Z., Lee K.F., Lemke G., Kilpatrick T.J. (1996). Cell death in the Schwann cell lineage and its regulation by neuregulin. Proc. Natl. Acad. Sci. USA.

[69] Sandrock A.W., Dryer S.E., Rosen K.M., Gozani S.N., Kramer R., Theill L.E., Fischbach G.D. (1997). Maintenance of acetylcholine receptor number by neuregulins at the neuromuscular junction in vivo. Science.

[70] Loeb J.A., Khurana T.S., Robbins J.T., Yee A.G., Fischbach G.D. (1999). Expression patterns of transmembrane and released forms of neuregulin during spinal cord and neuromuscular synapse development. Development.

[71] Módol-Caballero G., Herrando-Grabulosa M., García-Lareu B., Solanes N., Verdés S., Osta R., Francos-Quijorna I., López-Vales R., Calvo A.C., Bosch A. (2020). Gene therapy for overexpressing Neuregulin 1 type I in skeletal muscles promotes functional improvement in the SOD1 ALS mice. Neurobiol. Dis..

[72] Ueta R., Sugita S., Minegishi Y., Shimotoyodome A., Ota N., Ogiso N., Eguchi T., Yamanashi Y. (2020). Gene Therapy Enhances Neuromuscular Junction Innervation and Motor Function in Aged Mice. iScience.

[73] Zhou J.S., Li A., Li X.J., Yi J.X. (2019). Dysregulated mitochondrial Ca and ROS signaling in skeletal muscle of ALS mouse model. Arch. Biochem. Biophys..

[74] Bernardini C., Censi F., Lattanzi W., Barba M., Calcagnini G., Giuliani A., Tasca G., Sabatelli M., Ricci E., Michetti F. (2013). Mitochondrial Network Genes in the Skeletal Muscle of Amyotrophic Lateral Sclerosis Patients. PLoS ONE.

[75] Russell A.P., Wada S., Vergani L., Hock M.B., Lamon S., Léger B., Ushida T., Cartoni R., Wadley G.D., Hespel P. (2013). Disruption of skeletal muscle mitochondrial network genes and miRNAs in amyotrophic lateral sclerosis. Neurobiol. Dis..

[76] Bozzo F., Mirra A., Carrì M.T. (2017). Oxidative stress and mitochondrial damage in the pathogenesis of ALS: New perspectives. Neurosci. Lett..

[77] Halter B., de Aguilar J.L.G., Rene F., Petri S., Fricker B., Echaniz-Laguna A., Dupuis L., Larmet Y., Loeffler J.P. (2010). Oxidative stress in skeletal muscle stimulates early expression of Rad in a mouse model of amyotrophic lateral sclerosis. Free. Radic. Biol. Med..

[78] Méndez-López I., Sancho-Bielsa F.J., Engel T., García A.G., Padín J.F. (2021). Progressive Mitochondrial SOD1 Accumulation Causes Severe Structural, Metabolic and Functional Aberrations through OPA1 Down-Regulation in a Mouse Model of Amyotrophic Lateral Sclerosis. Int. J. Mol. Sci..

[79] Yoshino H. (2019). Edaravone for the treatment of amyotrophic lateral sclerosis. Expert. Rev. Neurother..

[80] Cha S.J., Kim K. (2022). Effects of the Edaravone, a Drug Approved for the Treatment of Amyotrophic Lateral Sclerosis, on Mitochondrial Function and Neuroprotection. Antioxidants.

[81] Liu H., Feng Y., Xu M., Yang J., Wang Z.C., Di G.F. (2018). Four-octyl itaconate activates Keap1-Nrf2 signaling to protect neuronal cells from hydrogen peroxide. Cell Commun. Signal.

[82] Zhou Y.J., Tang J.S., Lan J.Q., Zhang Y., Wang H.Y., Chen Q.Y., Kang Y.Y., Sun Y., Feng X.H., Wu L. (2023). Honokiol alleviated neurodegeneration by reducing oxidative stress and improving mitochondrial function in mutant SOD1 cellular and mouse models of amyotrophic lateral sclerosis. Acta Pharm. Sin. B.

[83] Liu X.L., Zhang J., Li J., Song C.J., Shi Y.G. (2022). Pharmacological inhibition of ALCAT1 mitigates amyotrophic lateral sclerosis by attenuating SOD1 protein aggregation. Mol. Metab..

[84] Zhang Y.G., Wu S.P., Yi J.X., Xia Y.L., Jin D.P., Zhou J.S., Sun J. (2017). Target Intestinal Microbiota to Alleviate Disease Progression in Amyotrophic Lateral Sclerosis. Clin. Ther..

[85] Li A., Li X.J., Yi J.X., Ma J.J., Zhou J.S. (2021). Butyrate Feeding Reverses CypD-Related Mitoflash Phenotypes in Mouse Myofibers. Int. J. Mol. Sci..

[86] Li X.J., Dong L., Li A., Yi J.X., Brotto M., Zhou J.S. (2022). Butyrate Ameliorates Mitochondrial Respiratory Capacity of The Motor-Neuron-like Cell Line NSC34-G93A, a Cellular Model for ALS. Biomolecules.

[87] Li A., Yi J.X., Li X.J., Dong L., Ostrow L.W., Ma J.J., Zhou J.S. (2024). Distinct transcriptomic profile of satellite cells contributes to preservation of neuromuscular junctions in extraocular muscles of ALS mice. eLife.

